# Blood-brain barrier permeability analysis of plant ceramides

**DOI:** 10.1371/journal.pone.0241640

**Published:** 2020-11-02

**Authors:** Koichi Eguchi, Daisuke Mikami, Hui Sun, Takuya Tsumita, Kaori Takahashi, Katsuyuki Mukai, Kohei Yuyama, Yasuyuki Igarashi

**Affiliations:** 1 Innovation and Business Development Headquarters, Daicel Corporation, Tokyo, Japan; 2 Lipid Biofunction Section, Faculty of Advanced Life Science, Hokkaido University, Sapporo, Hokkaido, Japan; 3 Department of Vascular Biology and Molecular Pathology, Hokkaido University Graduate School of Dental Medicine, Sapporo, Hokkaido, Japan; Hungarian Academy of Sciences, HUNGARY

## Abstract

Ceramides, a type of sphingolipid, are cell membrane components and lipid mediators that modulate a variety of cell functions. In plants, ceramides are mostly present in a glucosylated glucosylceramide (GlcCer) form. We previously showed that oral administration of konjac-derived GlcCer to a mouse model of Alzheimer’s disease reduced brain amyloid-β and amyloid plaques. Dietary plant GlcCer compounds are absorbed as ceramides, but it is unclear whether they can cross the blood-brain barrier (BBB). Herein, we evaluated the BBB permeability of synthetic plant-type ceramides (4, 8-sphingadienine, d18:2) using mouse and BBB cell culture models, and found that they could permeate the BBB both *in vivo* and *in vitro*. In addition, administrated ceramides were partially metabolized to other sphingolipid species, namely sphingomyelin (SM) and GlcCer, while crossing the BBB. Thus, plant ceramides can cross the BBB, suggesting that ceramides and their metabolites might affect brain functions.

## Introduction

Ceramides are a type of sphingolipid consisting of a sphingoid base and a fatty acid linked by an amide bond. Ceramides are major components of cell membranes, and they also function as signal mediators in cell differentiation, migration, and intracellular signal transduction [1–9]. The chemical structure of sphingolipid bases varies widely among species. Sphingosine (d18:1) is the most common in mammals, followed by sphinganine (d18:0) and phytosphingosine (t18:0), whereas 4,8-sphingadienine (d18:2) and 4-hydroxy-8-sphingadenin (t18:1) are most abundant in plant species [10]. In plants, ceramides are mostly present in a GlcCer glucosylated form. Orally administrated GlcCer are degraded to ceramide and glucose by specific enzymes in the digestive tract [11–13], and ceramide is subsequently broken down into a sphingoid base and a fatty acid [13]. Liberated sphingoid bases are incorporated into intestinal epithelial cells and reconstructed to ceramides from sphingoid bases, then absorbed into the body [14–16].

Alzheimer’s disease (AD) is a type of dementia that negatively impacts social life due to memory impairment and cognitive decline [17]. The pathology of AD begins with the formation of amyloid plaques (senile plaques) due to the accumulation of amyloid-β protein (Aβ) in the brain [18, 19]. In a previous study, we reported that oral administration of GlcCer extracted from *Amorphophallus Konjac* to an AD mouse model markedly decreased Aβ accumulation, suppressed amyloid deposition in the brain, and improved cognitive ability in a Y-maze learning task [20, 21]. In addition, after plant GlcCer administration, levels of neuronal exosomes, a type of extracellular vesicle (EV), were elevated in blood and brain tissues [21]. Our previous studies demonstrated that neuronal exosomes promote Aβ clearance, and plant ceramides enhance production of exosomes in neuronal cells [22, 23], suggesting that plant ceramides might affect exosome production within the brain.

As mentioned above, plant ceramides can be absorbed into the body, but it remains unclear whether they can enter the brain by permeating the blood-brain barrier (BBB). To determine the BBB permeability of plant ceramides, we synthesized a short-chain ceramide (d18:2/C5, d18:2/C6) and a long-chain ceramide (d18:2/C15) and used the synthetic ceramides to examine BBB permeability. We demonstrated that plant ceramides could permeate the BBB in both mouse and BBB cell culture models, and found that they were partially metabolized to SM and GlcCer while crossing the BBB.

## Materials and methods

### Chemicals and reagents

Fluorescein sodium, fluorescein isothiocyanate-dextran FD10S (FITC-dextran: average molecular weight 10,000), bovine serum albumin (BSA) were purchased from Sigma-Aldrich (St. Louis, MO, USA). Valeric anhydride, hexanoic anhydride, n-pentadecanoic acid, ethanol, and dodecane were purchased from Fujifilm Wako (Osaka, Japan). N-heptadecanoyl-D-*erythro*-sphingosine (C17 ceramide;d18:1/17:0), N-heptadecanoyl-D-*erythro*-sphingosylphosphorylcholine (C17 SM; d18:1/17:0), D-glucosyl-1, 1’-N-lauroyl-D-*erythro*-sphingosine (C12 GlcCer; d18:1/12:0) were purchased from Avanti Polar Lipids (Alabaster, Alabama, USA).

### Preparation of ceramides

Before synthesizing ceramides for assays, we analyzed amounts of plant sphingolipids in the medium of BBB culture model kit or mouse brains, and the ceramide species almost not included in the medium or brain were selected for assays. Plant ceramides used in the experiments were synthesized by binding fatty acids (C5 pentanoic acid, C6 hexanoic acid, C15 n-pentadecanoic acid) to sphingadienine (d18:2^4t8c^) to make the resulting products distinguishable from endogenous ceramides. The number of carbon atoms in fatty acids in the synthesized ceramides was selected to ensure that they were barely included in the cell medium and brain tissue.

4,8-Sphingadienine (d18:2^4t8c^) was prepared from the ethanol extract of konjac tuber (Daicel Corp., Osaka, Japan). Briefly, the ethanol extract of konjac tuber was incubated with chloroform: methanol: water (1:2:1) containing 0.4 M NaOH at 37°C for 2 h. Chloroform and water were added to the mixture to attain a 1:1:1 (v:v:v) ratio of chloroform: methanol: water. The lower phase containing GlcCer was collected, and the resulting lipids were re-extracted twice from the upper phase with chloroform. Extracted lipids were separated by silica gel column chromatography via gradient elution consisting of chloroform: methanol: acetic acid at 190:9:1→9:1:0→8:2:0 (v:v:v). Eluted lipids were monitored by thin-layer chromatography (TLC) analysis and fractions containing GlcCer were eluted with 8:2:0 (v:v:v) chloroform: methanol: acetic acid and collected. GlcCer were hydrolyzed in dioxane:10% Ba (OH)_2_ (1:1, v:v) at 110°C for 24 h [24]. Liberated sphingoid bases were purified by silica gel column chromatography eluted with chloroform: methanol:2 M ammonium hydroxide aqueous solution (40:10:1, v:v:v). The sphingoid base d18:2^4t8c^ was finally isolated by high-performance liquid chromatography (HPLC) with a CAPCELL PAK C18 MG column (particle size 5 μm; diameter 4.6 mm; length 250 mm; SHISEIDO, Tokyo, Japan) eluted with methanol:5 mM KH2PO4-K2HPO4 buffer pH 7.5 (85:15, v:v) at 1 mL/min and detected by measuring the UV absorbance at 210 nm.

Plant ceramides d18:2^4t8c^/5:0 (d18:2/C5) and d18:2^4t8c^/6:0 (d18:2/C6) were synthesized by the condensation reaction with 4,8-sphingadienine (d18:2^4t8c^) and their respective carboxylic anhydride under methanol. d18:2^4t8c^/C15:0 (d18:2/C15) was synthesized by the condensation reaction with 4,8-sphingadienine (d18:2^4t8c^) and n-pentadecanoic acid under tetrahydrofuran, 1-hydroxybenzotriazole monohydrate, and 1-ethyl-3-(N,N’-dimethylaminopropyl) carbodiimide hydrochloride. Synthesized crude plant ceramides were purified by HPLC, dissolved in 2% dodecane/98% ethanol to 10 mM, and used for *in vitro* and *in vivo* experiments.

### Animals and treatments

BALB/cCrSlc mice (10 weeks old) were purchased from Japan SLC (Hamamatsu, Japan). All animals were maintained in barrier facilities, housed in a room kept at 23 ± 1°C with a 12 h light/dark cycle, and allowed free access to tap water and normal diet food. Animal protocols were approved by the animal care committees of Hokkaido University, and all experiments were performed in accordance with guidelines and regulations of the animal care committees of Hokkaido University.

### *In vivo* BBB permeability assay

A 100 μL volume of 0.1 mM or 1 mM each ceramide (or vehicle only for controls) and 2 mM FITC-dextran dissolved in 1% BSA/phosphate-buffered saline (PBS) was injected into BALB/cCrSlc mice via the tail vein. Before injection, we mixed sample solutions to disperse by vortex and sonication, and incubated at 37°C for 10 min. To measure the concentration of ceramides in brain tissues, mice were perfused with PBS containing 5 U/mL heparin at 30 min after injection, and brain tissues were collected.

Isolated brains were suspended in PBS and sonicated with an ultrasonic homogenizer (TAITEC, Saitama, Japan). The concentration of ceramides in brains was measured by liquid chromatography-tandem mass spectrometry (LC-MS/MS) analysis, and that of FITC-dextran was measured with a Appliskan fluorescence plate reader (Thermo Scientific, Waltham, MA). To analyze the blood profile of the injected ceramides and metabolites, we intravenously injected a 100 μL volume of 1 mM each ceramide under the same conditions as above, then dissected and collected blood from the mouse heart at 30 min after the injection. We obtained serum from the collected blood, and measured the ceramide concentration by LC-MS/MS.

### *In vitro* BBB permeability assay

To evaluate the *in vitro* BBB permeability of plant ceramides, a Rat BBB Culture Model Kit (PharmaCo-Cell Co. Ltd., Nagasaki, Japan), which consist of primary cultures of brain capillary endothelial cells, brain pericytes and astrocytes [25, 26], was used. In the BBB model, vascular endothelial cells are plated in the insert, pericytes under the bottom of the inserts, and astrocytes on the bottom of the well. This BBB model has various *in vivo* BBB features, and used for a variety of previous BBB researches [27–30]. These cells were cultured according to the manufacturer’s instructions for 4−7 days to form a BBB-like structure, and transendothelial electrical resistance (TEER) was measured just before starting BBB permeability assays to ensure that the electrical resistance was higher than 150 Ω × cm^2^. A 5 μM ceramide solution (or vehicle only for control) and 26.6 μM fluorescein sodium (impermeable chemical) dissolved in culture medium were added to the upper wells (200 μL, vascular side), and the conditioned medium in the bottom wells (900 μL, brain side) was collected at 30 min after addition. The concentration of ceramides in the bottom wells was measured by LC-MS/MS analysis, and the concentration of fluorescein sodium was measured with a fluorescence plate reader.

### Cellular apoptosis and necrosis assays

Following *in vitro* BBB permeability assay, the numbers of vascular endothelial cells were immediately counted by using Apoptotic/Necrotic/Healthy Cells Detection Kit (PromoCell, Heidelberg, Germany). Briefly, vascular endothelial cells on transwell insert membranes were washed twice with an isotonic buffer containing calcium. Then the cells were stained by FITC-Annexin V, Ethidium Homodimer III, and Hoechst 33342 at 25°C for 15 min. After the staining, the cells were washed twice with an isotonic buffer containing calcium. The membranes were cut off from transwell inserts, mounted on glass slide, and observed under BZ-X710 fluorescence microscope (KEYENCE, Osaka, Japan). The levels of each signal as number of cells were quantified by BZ-X analyzer (KEYENCE).

### Ceramide quantification by LC-MS/MS

Sample solutions (0.8 mL) were transferred into a screw-capped glass test tube and mixed with chloroform (2 mL), methanol (1 mL), and 0.25 nmol of internal standards (C17 ceramide, C12 GlcCer, C17 SM), and test tubes were incubated at 48°C for 2h in shakers. After incubation, 10 M KOH (0.15 mL) was added and mixed by vortexing, and test tubes were incubated at 37°C for 2 h in shakers. Chloroform (1 mL) and water (1 mL) were added, test tubes were centrifuged at 1000 × g for 5 min, and the lower phases were collected and placed in fresh test tubes. Chloroform (2 mL) was added to the upper phases, mixed by vortexing, test tubes were centrifuged at 1000 × g for 5 min, and the lower phases were re-collected, placed in fresh test tubes, dried under a nitrogen stream, and dissolved in 100 μL of acetonitrile: methanol (19:1, v:v) for analysis by LC-MS/MS.

LC-MS/MS analysis was carried out using a Triple TOF 5600 system (AB SCIEX, Foster City, CA, USA) in electrospray ionization (ESI)-positive mode. A 10 μL volume of sample solution was injected onto an InertSustain NH2 hydrophilic interaction chromatography column (particle size 5 μm; diameter 2.1 mm; length 100 mm; GL Science, Tokyo, Japan). Solvent A was acetonitrile: methanol: formic acid (95:5:0.2, v:v:v) containing 5 mM ammonium formate, while solvent B was methanol: formic acid (100:0.2, v:v) containing 5 mM ammonium formate. Samples were eluted at 0.2 mL/ min over a 45 min gradient (0−5 min 0% B, 5−10 min from 0% to 20% B, 10−12 min 20% B, 12–15 min from 20% to 50% B, 15−22 min 50% B, 22−27 min from 50% to 80% B, 27−30 min, 30–45 min from 80% to 0% B).

## Results

### Intravenously-injected plant-type ceramides enter mouse brains

**Fig 1:**
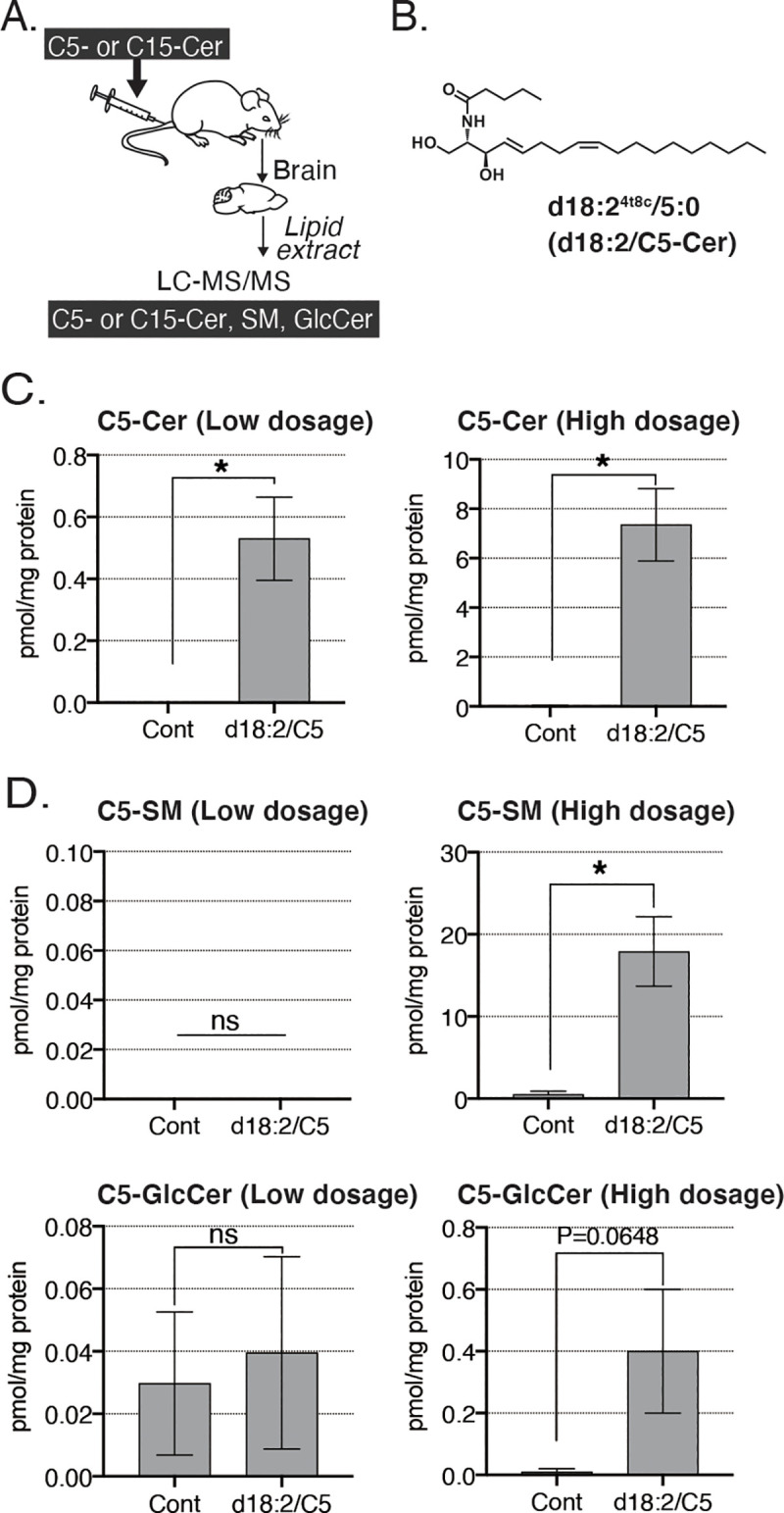
*In vivo* Blood-Brain Barrier (BBB) permeability of C5 ceramides. (A) Schematic representation of the *in vivo* assay. Synthetic plant-type ceramides were injected intravenously into mice. After 30 min, the brain was removed and analyzed by LC-MS/MS. (B) Chemical structure of d18:2/C5-ceramide. (C) Levels of d18:2/C5-ceramide in the brain after injection at low (0.1 mM) or high (1 mM) dosage. Data are presented as means ± standard deviation (SD; n = 3 per group; *p <0.05 by t-test). (D) Levels of d18:2/C5-SM and d18:2/C5-GlcCer in the brain after injection at low and high dosage. Data are presented as means ± SD (n = 3 per group; ns, not significant; *p <0.05 by t-test).

We examined whether exogenous plant-type ceramides d18:2/C5 and d18:2/C15 can enter mouse brains. First, we intravenously injected a low (0.1 mM) or high (1 mM) dosage of synthetic short-chain d18:2/C5 ceramides, then dissected and removed whole brains at 30 min after injection, and measured the ceramide concentration by LC-MS/MS (Fig 1A and 1B). Compared with the control group, the amount of d18:2/C5-ceramide in mouse brains was increased in the d18:2/C5-treated group at both low and high dosage, and this increase was dose-dependent (Fig 1C). Interestingly, while the levels of ceramide metabolites SM and GlcCer in mouse brains were not different between controls and the d18:2/C5-treated group receiving a low dosage, levels were clearly elevated in the d18:2/C5-treated group receiving a high dosage (Fig 1D).

**Fig 2:**
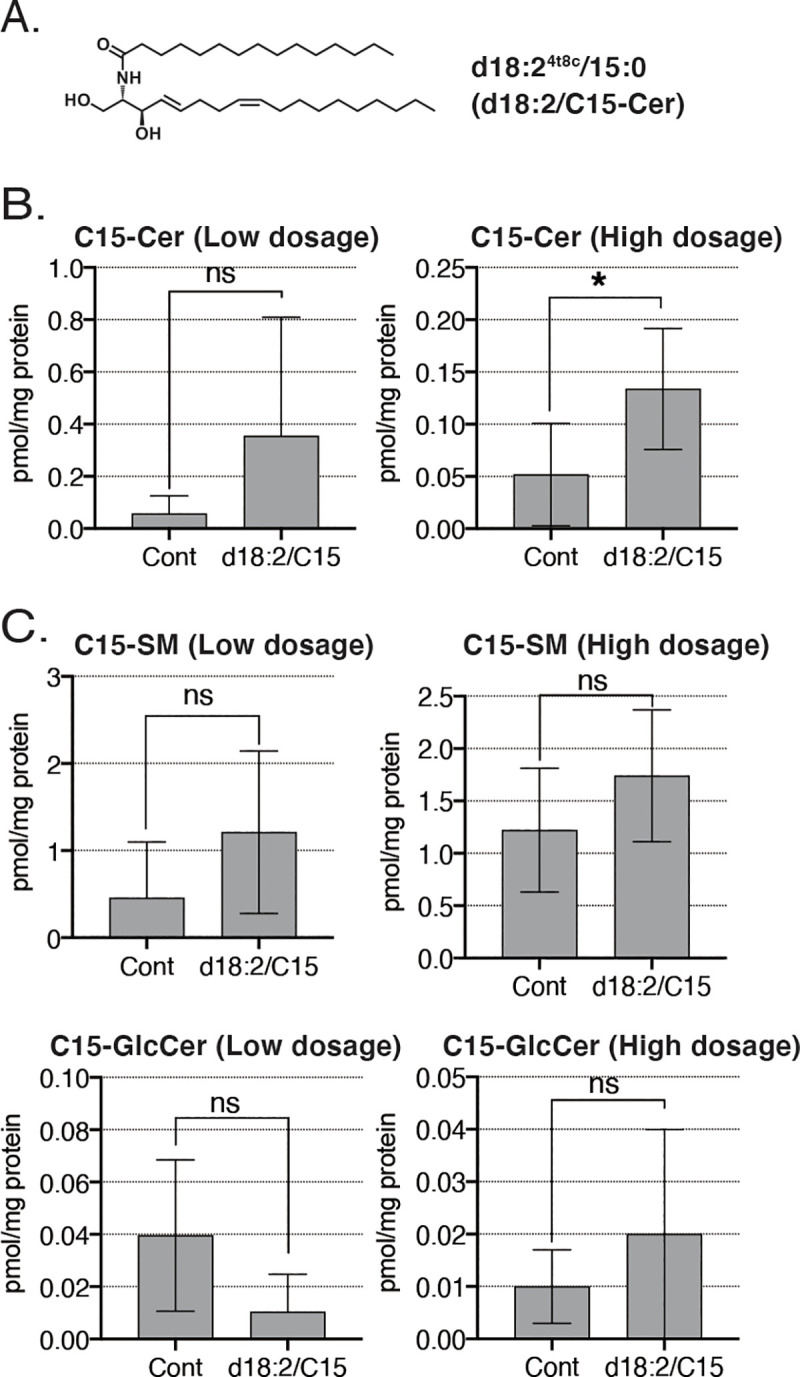
*In vivo* Blood-Brain Barrier (BBB) permeability of C15 ceramide. (A) Chemical structure of d18:2/C15-ceramide. (B) Levels of d18:2/C15-ceramide in the brain after injection at low and high dosage. Data are presented as means ± SD (Low dosage: n = 3, each group, High dosage: Control, n = 9; d18:2/C15, n = 8; ns, not significant; *p<0.05 by t-test). (C) Levels of d18:2/C15-SM and d18:2/C15-GlcCer in the brain after injection at low and high dosage. Data are presented as means ± SD (Low dosage: n = 3, each group, High dosage: Control, n = 9; d18:2/C15, n = 8; ns, not significant by t-test).

Next, we injected a low (0.1 mM) or high (1 mM) dosage of long-chain ceramides (d18:2/C15) into mice. At 30 min after injection, brains were collected, and ceramides concentrations were analyzed (Fig 2A). Compared with the control group, levels of d18:2/C15-ceramide in mouse brains were significantly increased in the d18:2/C15-treated group receiving a high dosage (Fig 2B). Although changes in SM and GlcCer levels were not statistically significant, they tended to increase in the d18:2/C15-treated group following a high dosage (Fig 2C). Passing rate of injected ceramides crossing the BBB was C5 low dosage: 2.43 mol%, C5 high dosage: 11.3 mol%, C15 high dosage: 0.28% (total molar amount of ceramide, SM, GlcCer in brains).

Our experiments confirmed that there was no difference in the fluorescence intensity of FITC-dextran between control and ceramide-treated groups (S1 Fig). Additionally, we verified whether administrated ceramides convert to SM and GlcCer in peripheral tissues. We collected the blood from mice injected ceramides (100 μL volume of 1 mM), and measured the ceramide concentration in serum by LC-MS/MS. Compared to the control group, the levels of ceramides and their metabolites, SM and GlcCer, increased in the d18:2/C5- or d18:2/C15-treated group. Especially, SM levels was the highest among them (S2 Fig). The order of their concentrations in serum correlates with that in brain tissues.

The results showed that plant-type ceramides can pass from circulating blood into brains through the BBB in mice. In addition, levels of ceramide metabolites GlcCer and SM were also increased in brains, raising the possibility that administrated ceramides may be converted to SM and GlcCer in the blood circulation, in the brain, or when crossing the BBB.

### Plant-type ceramides permeate the BBB in an *in vitro* model

**Fig 3:**
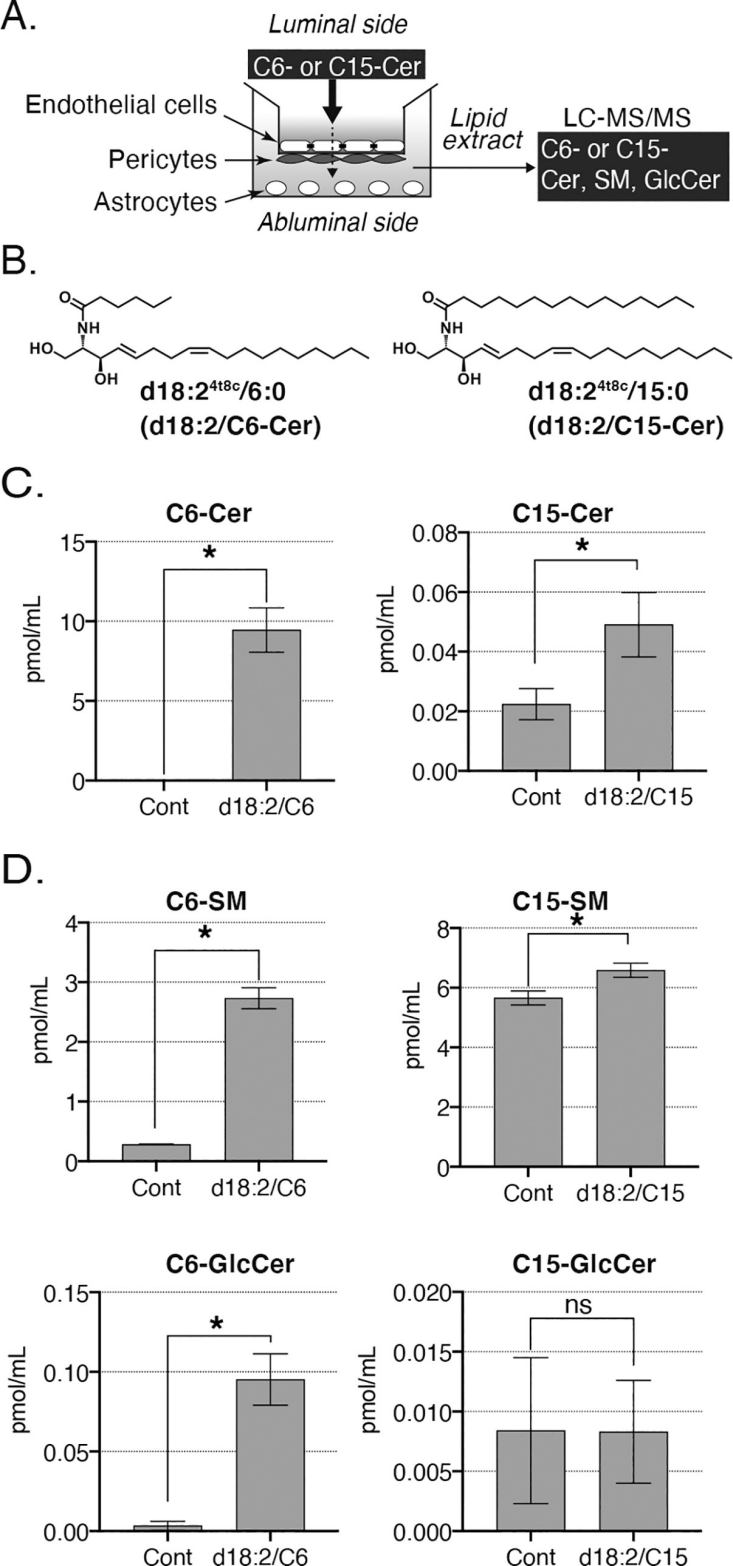
*In vitro* Blood-Brain Barrier (BBB) permeability. (A) Schematic representation of an *in vitro* BBB model constructed from primary cultures of rat brain capillary endothelial cells, brain pericytes, and astrocytes. Synthetic plant-type ceramides (5 μM) were added to the medium in the inserts. After 30 min, conditioned medium in the bottom wells was collected and analyzed by LC-MS/MS. (B) Chemical structure of d18:2/C5-ceramide. (B) Chemical structures of d18:2/C6- and d18:2/C15-ceramides. (C) Levels of d18:2/C6-ceramide and d18:2/C15-ceramide. Data are presented as means ± SD (n = 3; *p <0.05 by t-test. (D) Levels of d18:2/C6-SM, d18:2/C15-SM, and GlcCer. Data are represented as means ± SD (n = 3; ns, not significant; *p <0.05 by t-test).

To confirm the BBB permeability of plant-type ceramides, and to verify whether ceramides are metabolized while crossing the BBB, we used an *in vitro* BBB cell model that consists of primary cultures of brain capillary endothelial cells, brain pericytes, and astrocytes (Fig 3). In this experiment, we synthesized and employed d18:2/C6 plant-type ceramides as short-chain ceramides and d18:2/C15 as long-chain ceramides. We added each ceramide (5 μM) to the medium in the inserts (vascular side), and after 30 a min incubation we analyzed the ceramide concentration in the medium in the bottom of the wells (brain side; Fig 3A and 3B). Levels of both d18:2/C6-ceramide and d18:2/C15-ceramide were increased in both d18:2/C6- and d18:2/C15-treated groups (Fig 3C). Similarly, levels of d18:2/C6- and d18:2/C15-SM were also increased in both ceramide-treated groups (Fig 3D). However, GlcCer levels were increased in the d18:2/C6-ceramide-treated group, but not in the d18:2/C15-ceramide-treated group (Fig 3D). There was no difference in the fluorescence intensity of fluorescein sodium between control and ceramide-treated groups (S3 Fig). It is reported that ceramides, especially composed of short chain fatty acid, can induce apoptosis [31, 32]. We verified whether added ceramides induce apoptosis or necrosis of vascular endothelial cells that mainly construct the BBB [33] by staining with FITC-Annexin V, Ethidium Homodimer III, and Hoechst 33342. All signals showed no difference between control and the group, which had been treated with 5 or 50 μM ceramides (S4 Fig). In agreement with previous *in vivo* results, these findings showed that plant-type ceramides can permeate the BBB. Furthermore, the administrated ceramides were partially converted to other sphingolipid species while crossing the BBB.

## Discussion

The results of this study showed that plant-type ceramides can permeate the BBB in both *in vitro* and *in vivo* assays. In addition, we also found that the administrated ceramides were partially metabolized to SM and GlcCer, and these sphingolipid species accumulated within the brain.

Orally administered GlcCer are digested by glucocerebrosidase and ceramidase in the digestive tract, liberated sphingoid bases are transported into small intestine epithelial cells, and sphingolipid metabolites are resynthesized into ceramides, SM, GlcCer, and other compounds [14–16, 34]. In this study, we focused on measuring intact ceramides and two metabolites, SM and GlcCer, in the brain side to analyze the BBB permeability of the ceramides. When substances cross the BBB, they normally pass via cells that form the neurovascular unit (NVU) such as vascular endothelial cells, pericytes, and astrocytes [35]. The results of our *in vivo* BBB permeability assay demonstrated that administrated ceramides converted to SM and GlcCer in peripheral tissues in the blood circulation, it was suggested that they pass through the BBB as ceramides and their metabolites (Figs 1 and 2, S2 Fig). On the other hand, the results of *in vitro* BBB permeability assays suggested that the sphingolipid synthesis enzymes sphingomyelin synthase and glucosylceramide synthase metabolized the ceramides into SM and GlcCer within the NVU component cells (Fig 3). At present, there are two possible ways that arrow the ceramide metabolites to pass the BBB and further research is needed to clarify how much each way contributes their BBB permeability *in vivo*. In addition, other products derived from the injected ceramides, such as 4,8-sphingadienine and complex glycosphingolipids, might be also accumulated in the brains. Furthermore, the accumulated plant-type sphingolipids might affect intrinsic sphingolipids. In skins, it is reported that orally administered GlcCer stimulated *de novo* sphingolipid synthesis [16]. A challenging study in the future will be determined the effects of plant ceramide administration on the comprehensive profile of sphingolipids in the brains.

It is reported that sphingosine-1-phosphate (S1P), a lyso-type sphingolipid metabolite, changes the barrier function of the BBB by altering the localization of tight junction-related proteins such as VE-cadherin, claudin-5, and β-catenin [36]. By contrast, the present results indicate that the administrated ceramides did not affect the functional integrity or permeability of the BBB in experiments using the impermeable chemicals fluorescein sodium and FITC-dextran (S1 and S3 Figs).

These results also revealed that the BBB permeability rates of d18:2/C5 and d18:2/C6 were higher than d18:2/C15 (Figs 1C, 2B and 3C). The number of carbons in the fatty acid part of ceramides is believed to be involved in determining the BBB permeability rate because ceramides with longer fatty acids are less hydrophilic. Another possibility is that specific BBB transporters may be involved in ceramide passing. It has not yet reported been whether there are dedicated BBB transporters for specific ceramides, but Mfsd2a, a member of the major facilitator superfamily, is known to be transporter specific for lyso-SM [37]. In addition, a sphingolipid-binding protein, the COPI machinery protein p24, can distinguish the fatty acid length of SM, and bind specifically to C18-SM [38]. Thus, unknown transporters expressed in the BBB might distinguish the fatty acid length of ceramides and modulate their permeability.

In this study, we demonstrated that plant ceramides can enter the brain through the BBB to assay with *in vitro* BBB model and intravenous administration for mouse. These results suggest that the plant ceramides, which are administered parentally and absorbed as dietary materials, might affect brain functions crossing the BBB.

## Supporting information

S1 Fig The levels of fluorescence intensity in brains (*in vivo* permeability assay). A) low dosage: n = 3, each group. B) high dosage: None n = 3, Control n = 9, d18:2/C5 n = 3, d18:2/C15 n = 8. Data are presented as means ± SDs. ns, not significant; *P < 0.05 by t-test.
(TIF)

S2 Fig The levels of administrated ceramides and metabolites in the plasma (*in vivo* permeability assay). A) Levels of d18:2/C5-ceramide, d18:2/C5-SM, d18:2/C5-GlcCer in the plasma after injection at high dosage. (n = 4, each group. Data are presented as means ± SDs. ns, not significant; *p<0.05 by t-test). (B) Levels of d18:2/C15-ceramide, d18:2/C15-SM, d18:2/C15-GlcCer in the plasma after injection at high dosage. (n = 4, each group. Data are presented as means ± SDs. ns, not significant; *p<0.05 by t-test).
(TIF)

S3 Fig The levels of fluorescence intensity in the conditioned mediums (*in vitro* BBB model permeability assay). n = 3, each group. Data are presented as means ± SDs. ns, not significant; *P < 0.05 by t-test.
(TIF)

S4 Fig The levels of FITC-Annexin V, Ethidium Homodimer III, Hoechst 33342 signal in the conditioned vascular endothelial cells (*in vitro* BBB model permeability assay). (A) FITC-Annexin V stains the exposure of phosphatidylserine on the cell surfaces in apoptosis cells. Ethidium Homodimer III stains the exposure of DNA in necrosis cells. Hoechst 33342 stained the DNA inside cell in healthy cells. (B) n = 6, each group. Data are presented as means ± SDs. ns, not significant; *P < 0.05 by t-test.
(TIF)
