# Supplementary figures and images for: Blood-brain barrier permeability analysis of plant ceramides
Source: PLoS One. 2020 Nov 2;15(11):e0241640. doi: 10.1371/journal.pone.0241640 (PMC7605672; doi:10.1371/journal.pone.0241640)

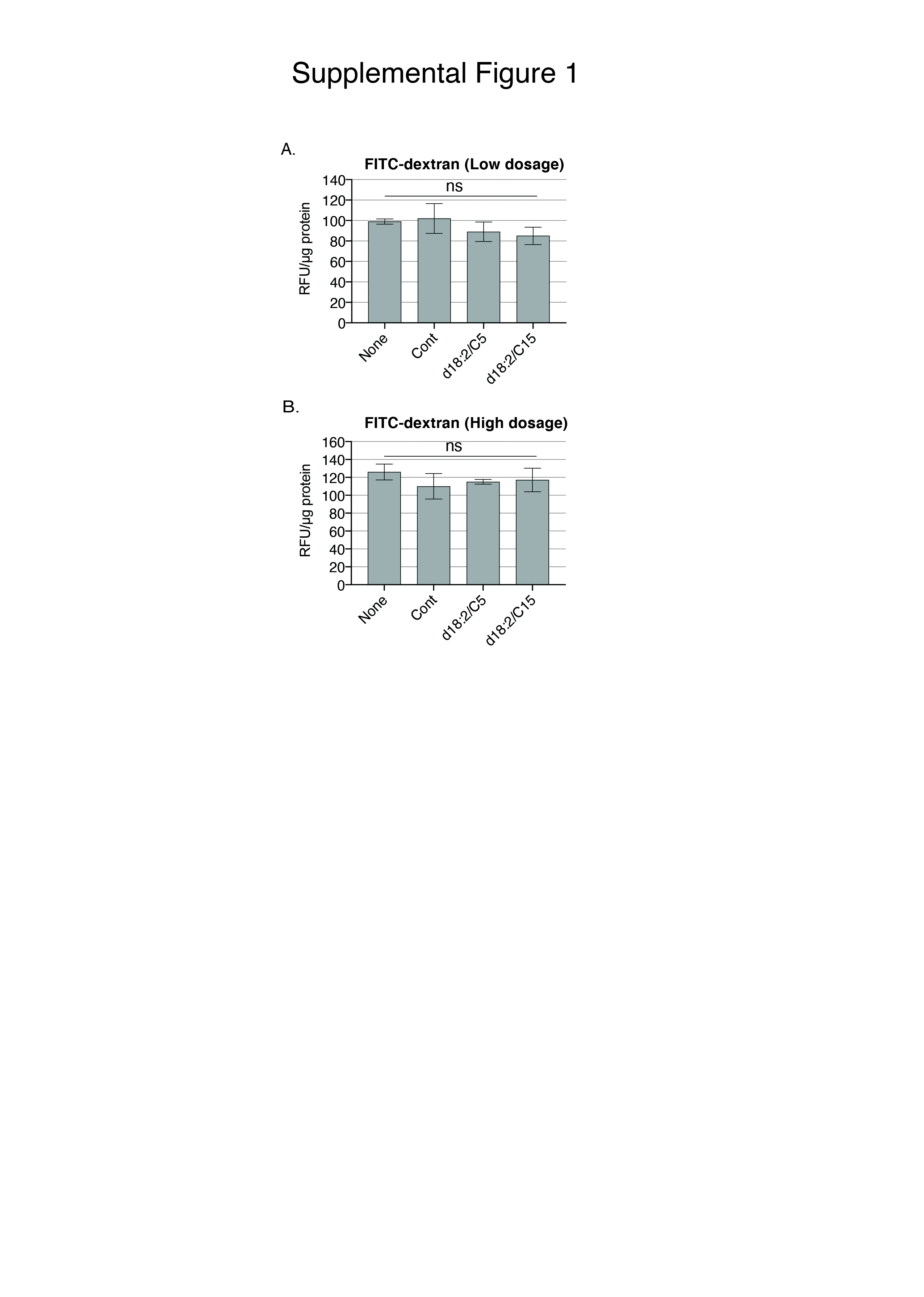

Supplement: S1 Fig — A) low dosage: n = 3, each group. B) high dosage: None n = 3, Control n = 9, d18:2/C5 n = 3, d18:2/C15 n = 8. Data are presented as means ± SDs. ns, not significant; *P < 0.05 by t-test. (TIF) [file pone.0241640.s001.tif]

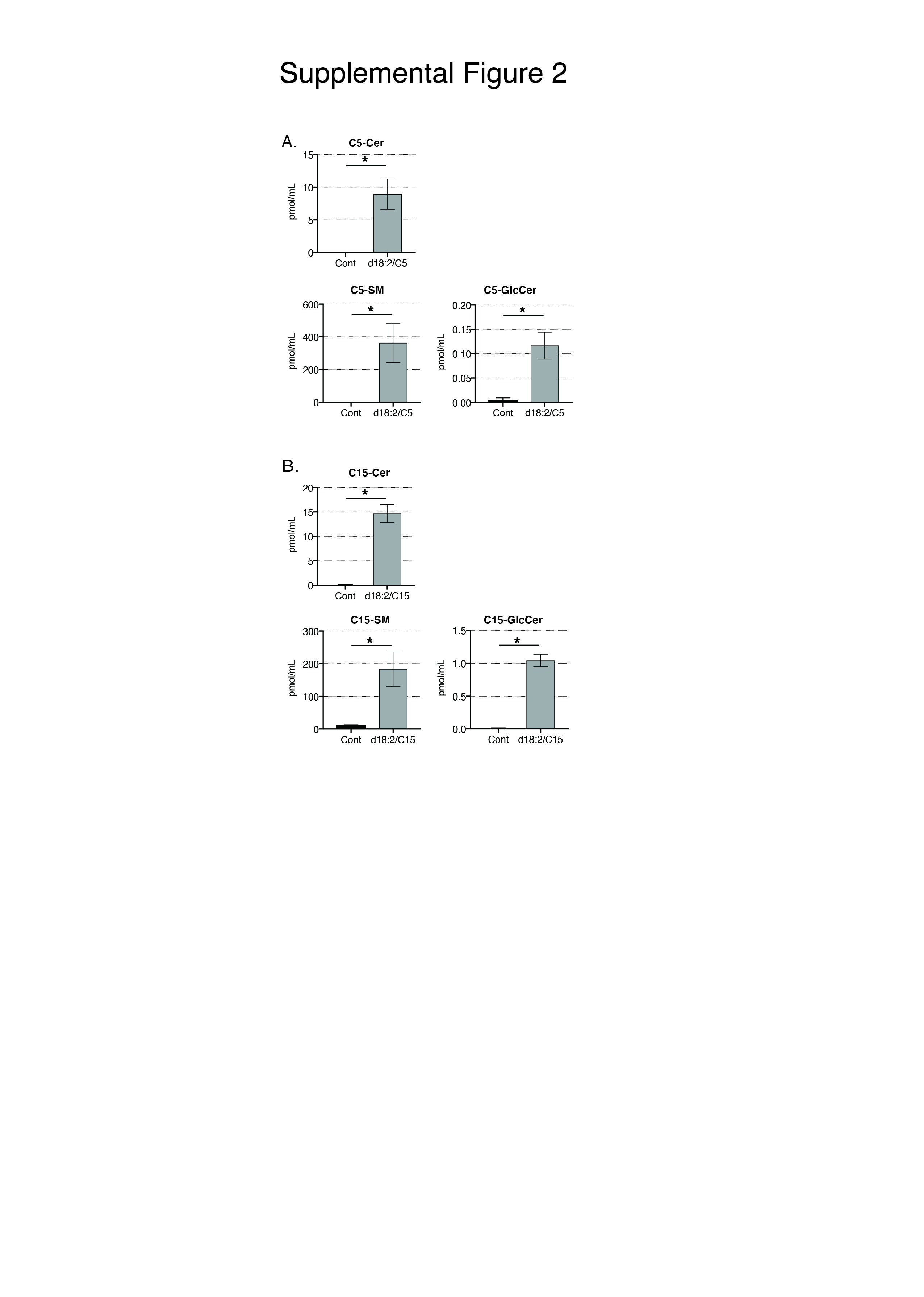

Supplement: S2 Fig — A) Levels of d18:2/C5-ceramide, d18:2/C5-SM, d18:2/C5-GlcCer in the plasma after injection at high dosage. (n = 4, each group. Data are presented as means ± SDs. ns, not significant; *p<0.05 by t-test). (B) Levels of d18:2/C15-ceramide, d18:2/C15-SM, d18:2/C15-GlcCer in the plasma after injection at high dosage. (n = 4, each group. Data are presented as means ± SDs. ns, not significant; *p<0.05 by t-test). (TIF) [file pone.0241640.s002.tif]

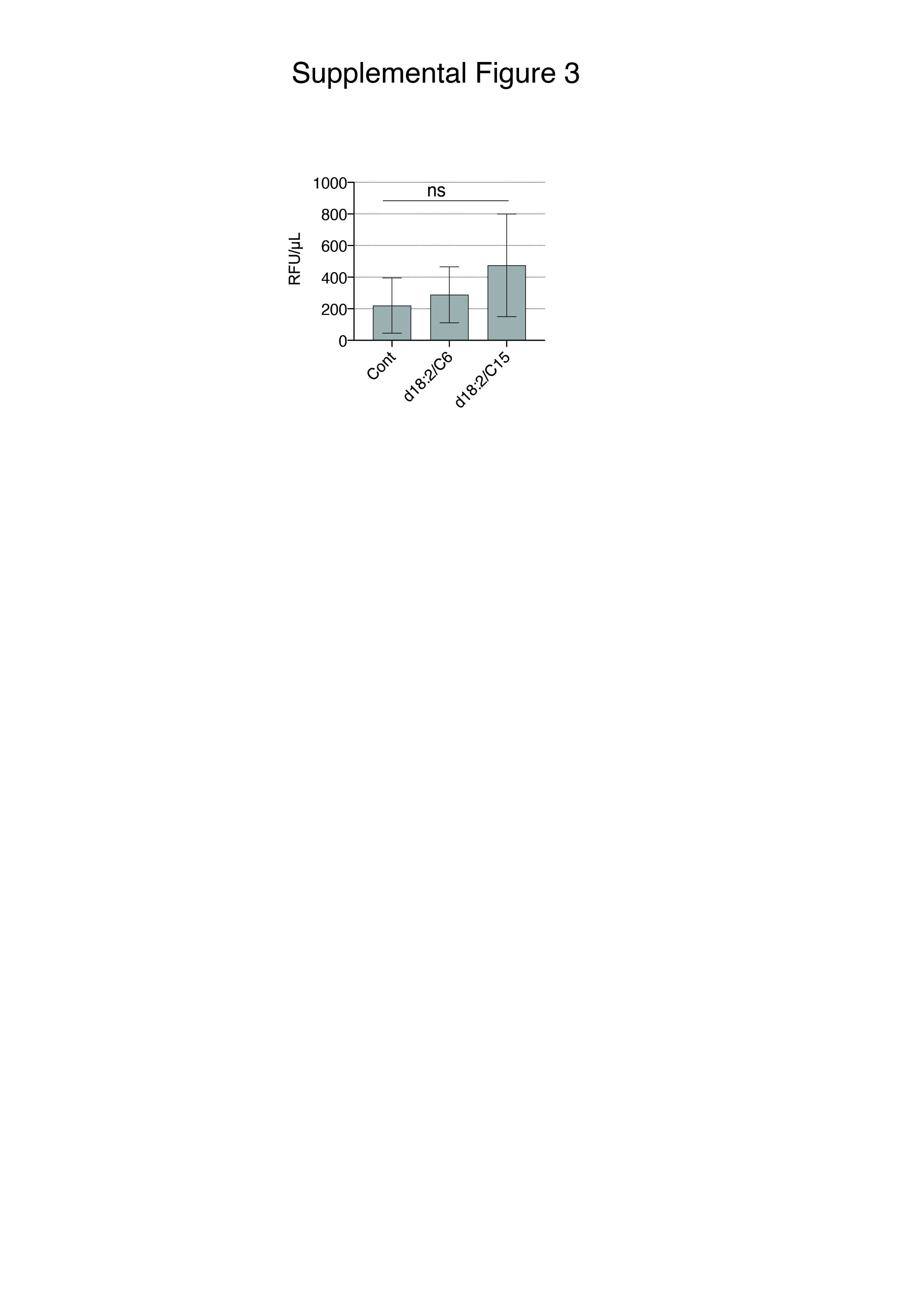

Supplement: S3 Fig — n = 3, each group. Data are presented as means ± SDs. ns, not significant; *P < 0.05 by t-test. (TIF) [file pone.0241640.s003.tif]

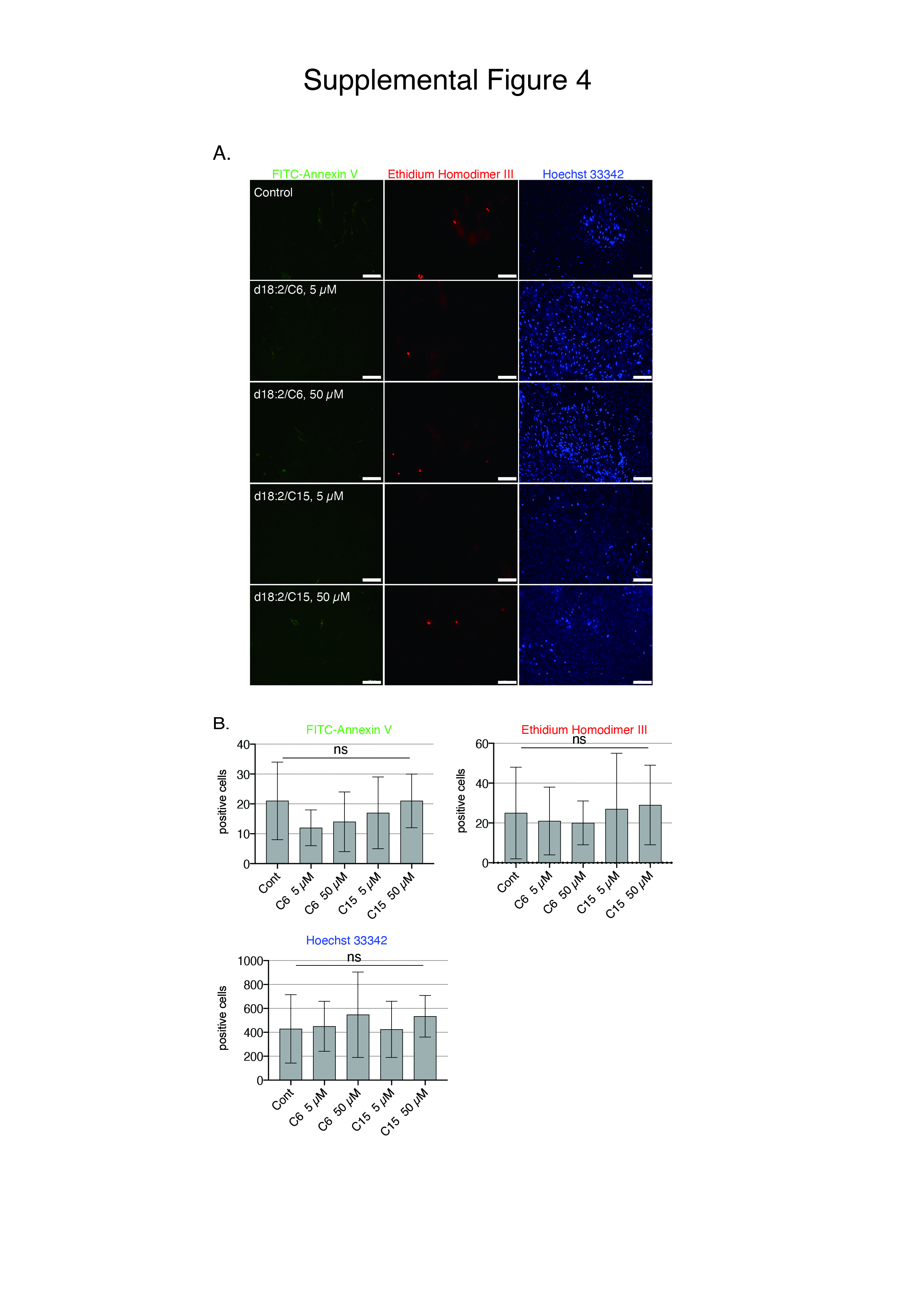

Supplement: S4 Fig — (A) FITC-Annexin V stains the exposure of phosphatidylserine on the cell surfaces in apoptosis cells. Ethidium Homodimer III stains the exposure of DNA in necrosis cells. Hoechst 33342 stained the DNA inside cell in healthy cells. (B) n = 6, each group. Data are presented as means ± SDs. ns, not significant; *P < 0.05 by t-test. (TIF) [file pone.0241640.s004.tif]
